# Metabolomic insights into inter-familial and pre-transport treatment effects on live transport stress in selectively-bred mussels, *Perna canaliculus*

**DOI:** 10.1007/s11306-026-02477-7

**Published:** 2026-06-16

**Authors:** M. C. F. Cheng, L. N. Zamora, N. J. Delorme, N. L. C. Ragg, A. J. R. Hickey, B. J. Dunphy

**Affiliations:** 1 School of Biological Sciences, University of Auckland, Private Bag 92019, Auckland, 1142 New Zealand; 2 Cawthron Institute, Private Bag 2, Nelson, 7042 New Zealand

**Keywords:** Selective breeding, Metabolomics, Climate change, Stress mitigation, Mussel, Aquaculture, Food security

## Abstract

**Introduction:**

Selectively-bred green-lipped mussel, *Perna canaliculus*, families may differ in their physiological responses, potentially altering their sensitivity to preparatory treatment and thereby influencing its effectiveness in mitigating transport stress for live transport.

**Objective:**

This study investigated whether selectively-bred mussel families differ in their metabolic responses to magnesium chloride (MgCl_2_) pre-treatment for mitigating live transport stress and enhancing recovery.

**Methods:**

Two full-sibling mussel families with contrasting physiological phenotypes, including a less thermally tolerant family (FamC) and a more thermally tolerant family (FamF), were subjected to MgCl_2_ pre-treatment followed by 72-hour simulated live transport and 5-day recovery. Gill metabolic profiles were analysed immediately after transport and during recovery using GC-MS.

**Results:**

MgCl_2_ pre-treatment reduced, but did not eliminate, transport-induced metabolic changes with family-specific responses observed. The less tolerant FamC showed stronger anaerobic metabolic responses to transport stress with 25 differentially expressed metabolites (DEM), resulting in 10 enriched pathways immediately after transport indicating a rapid but energetically costly stress response. In contrast, the more tolerant FamF showed a weaker transport response (12 DEM; 5 pathways) but activated broader metabolic pathways during recovery (21 DEM; 10 pathways), including metabolites linked to cellular protection after 1 day of recovery suggesting greater stress resilience and recovery capacity.

**Conclusion:**

The contrasting metabolic responses between mussel families indicate differences in transport stress resilience and recovery capacity, suggesting that selective breeding could improve resilience and pre-treatments effectiveness during live transport. Such pre-treatment could also be used for research and analytical purposes where minimising transport-induced physiological stress could improve sample quality and experimental reliability.

**Supplementary Information:**

The online version contains supplementary material available at https://doi.org/10.1007/s11306-026-02477-7.

## Introduction

The New Zealand green lipped mussel, *Perna canaliculus* (Gmelin 1791), is an important aquaculture species, which generates more than NZ$300 million annually (Aquaculture New Zealand, 2023). This species is, however, challenged by highly fluctuating environments in New Zealand, which has been liked to reduced aquaculture productivity and, in extreme cases, mass mortality events, such as the 2018 marine heatwave that caused substantial losses of *P. canaliculus* in commercial farms (Heasman et al., 2020; Newton & Webb, 2019; Pinkerton, 2017). Not all individuals are, however, affected equally during such events, with some mussels showing greater resilience and survival capacity than others. Individuals within the same species can vary greatly in their ability to tolerate environmental stress, reflecting underlying genetic diversity (Cheng et al. 2025a; Williams-Simon et al. 2024). These intrinsic inter-individual variation provide an opportunity to enhance stock performance through selective breeding (Ding et al., 2020; Ericson et al., 2024; Symonds et al., 2019). The aquaculture industry increasingly applies selective breeding programmes as an adaptive strategy to produce edible bivalves that are both highly productive under farm conditions and resilient to environmental extremes, such as marine heatwaves (Tan et al., 2020). Such practice helps develop mussels with desired traits, such as faster growth, better product quality and improved thermal tolerance, which are important for maintaining productivity under likely future extremes and increasingly fluctuating environments. The selective breeding programme for *P. canaliculus* has been operating for over two decades and has successfully developed family lines selected for desirable production traits, such as growth rate, resilience to environmental stress and overall survival under farming conditions (Camara & Symonds, 2014; Symonds et al., 2018).

Beyond on-farm performance, maintaining product quality of *P. canaliculus* along the supply chain is critical, especially with growing market demand for high-quality products derived from this mussel species, such as extracts, powder, frozen, and live export (Abshirini et al., 2021; Aquaculture New Zealand, 2023; Lomiwes et al., 2023). During live export, mussels are subjected to stressors including air exposure, fluctuating temperatures and mechanical disturbance (Harding et al., 2004; Barrento & Powell, 2016). These stressors can impair mussels’ physiological condition, reduce meat quality, and even lead to mortality (Andrade et al., 2018; De Andrade et al., 2021; Nguyen et al., 2020). These issues are particularly problematic in long-distance transport out of water, where stress accumulates over time. To address these challenges, pre-treatment strategies are being explored to condition mussels before transportation, aiming to increase their resilience during handling and transit from hatchery to farming site and/or from harvest to final market destination (e.g., Cheng et al. 2025b; Delorme et al. 2024a). These pre-treatments are designed to reduce metabolic activity or enhance stress tolerance, helping maintain quality and survival throughout the supply chain (e.g., Pozhoth & Jeffs, 2022; Tuckey et al., 2023). The effectiveness of the pre-treatment prior to live transport can be reflected in the mussels’ stress levels, as indicated by biomarkers, such as metabolites like succinate and alanine, and antioxidant enzymes (e.g., Delorme et al. 2024a; Dunphy et al. 2015). An effective pre-treatment would reduce stress, so mussels use less energy for stress management, which in turn would result in lower concentrations of these biomarkers (Dunphy et al., 2018). To determine whether a pre-treatment is effective across mussels with markedly different genetic backgrounds, however, a more precise and diagnostic approach is needed.

Metabolic profiling is a powerful approach used in aquaculture to evaluate physiological and stress responses of farmed species along the supply chain (Alfaro & Young, 2016). Metabolomics offers a detailed view of changes in metabolites associated with stress and recovery, revealing shifts in energy balance and protective mechanisms that help organisms cope with stress (e.g., Alfaro et al., 2021; Xu et al., 2021). Importantly, metabolomics enables an untargeted and comprehensive assessment of metabolic changes, capturing both pre-defined biomarkers and previously uncharacterised metabolites, since stress responses involve coordinated changes across multiple metabolic pathways, where different metabolites could contribute distinct physiological functions (Jiang et al., 2020). By examining metabolic profiles after transport stress and recovery, variation in stress responses can be assessed, providing a basis for evaluating the effectiveness of the pre-treatment. This approach helps determine whether the pre-treatment is effective across mussels exhibiting different phenotypes as a result of the selective breeding process. Integrating selective breeding with pre-transport conditioning and metabolic profiling provides a comprehensive approach to understand and compare different management strategies to improve mussel quality at market. This combined approach supports not only improved on-farm performance but also higher product quality and better stress resilience throughout the production and distribution stages, ensuring the stable supply chain in shellfish aquaculture.

Chemical pre-treatments have been investigated as a way to lower shellfish metabolism during live transport (e.g., Cheng et al., 2024, 2025a, 2025b; Pozhoth & Jeffs, 2022; Willis et al., 2024). For instance, pre-treatment of the Manila clam, *Ruditapes philippinarum*, with sodium nitroprusside reduced stress responses during 3-day aerial exposure by mitigating oxidative stress (Zheng et al., 2023). Sodium nitroprusside, however, poses food safety concerns due to cyanide formation during its metabolism (Friederich & Butterworth, 1995). The use of food-safe chemicals as pre-treatments should, therefore, be carefully considered for application in live shellfish transport destined for human consumption (e.g., Pozhoth & Jeffs, 2022). Magnesium chloride (MgCl_2_), a food-safe metabolic suppressant relatively widely used in seafood processing with minimal safety concerns (EFSA-FAF, 2019) has been explored as a treatment to reduce stress associated with post-harvest handling in mussels (Cheng et al. 2025b), and its potential as a pre-treatment to alleviate stress during mussel live transport is currently under investigation (Cheng et al., 2026).

The relationship between family-level variation and the effectiveness of MgCl_2_ in decreasing mussel metabolism remains unclear, as differences among mussel families may influence their sensitivity to a given chemical dose. Given that selectively-bred mussel families may differ in physiological traits associated with stress tolerance and metabolic regulation, it is plausible that their responses to a given chemical dose may also vary. It is, however, still unclear whether such differences actually influence sensitivity to MgCl_2_. This study, therefore, aimed to determine whether the efficacy of MgCl_2_ pre-treatment differs between selectively-bred mussel families. Mussels were sourced from an established breeding programme in which families had been selected to include traits such as growth rate and meat yield (e.g., Camara & Symonds, 2014; Ibarrola et al., 2017). Nonetheless, the families used in this study were also used in a previous study investigating the effects of genetics and age on thermal tolerance (Delorme et al. 2024b). It was, therefore, important to evaluate if MgCl_2_ pre-treatment has consistent effectiveness across families in terms of metabolic responses during live transport and subsequent recovery processes. Answering these questions will help determine the applicability of MgCl_2_ as a generic strategy for improving the quality and resilience of mussels during live transport and considers the opportunities to breed for improved performance to market.

## Materials and methods

### Source of mussels and maintenance

**Fig. 1:**
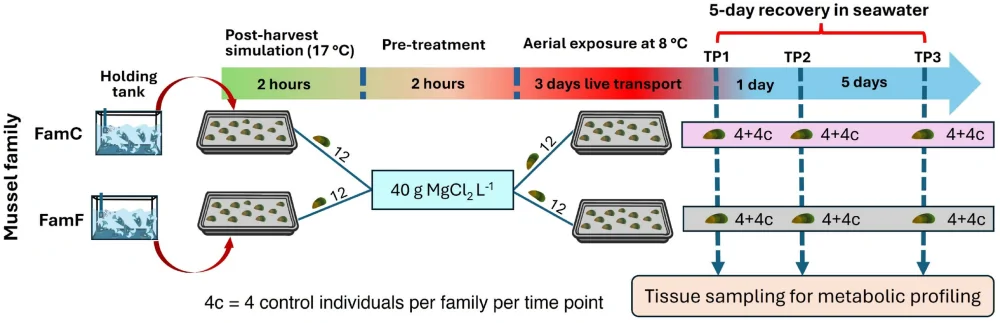
Diagram illustrating the experimental design for the mussel live transport simulation and recovery study. The experiment was conducted in two identical rounds, with each round involving gill tissue sampling from four mussels from each of the selected families and four control mussels per family from holding tanks for each time point (i.e., control mussels were not subjected to either the MgCl_2_ treatment or the live transport simulation). This resulted in a total of eight mussels per treatment and control group for each family across both rounds at each time point. Sampling was performed at three time points: TP1 (after 72-hour live transport simulation), TP2 (after 1 day of recovery in seawater), and TP3 (after 5 days of recovery in seawater)

Mussels (shell length: 55–99 mm, three years old) used in this experiment were produced through a selective breeding programme in 2021, where various families were established using single-parent matings (Delorme et al. 2024b). The main goal of this breeding programme was to produce different mussel families with desirable traits such as improved growth and higher meat quality. Hatchery-bred juvenile mussels were transferred to a mussel farm in the Marlborough Sounds, South Island, New Zealand for grow-out. After 52 weeks, mussels from six selected families were collected from the farm and transported to the Cawthron Aquaculture Park (CAP) for heat tolerance assessments in 2022 (see Delorme et al. 2024b for more details). The mussels were subsequently maintained in indoor holding tanks at CAP, with flow-through (~ 3 L/min) seawater (10–18 °C, salinity ~ 34–35, reflecting natural seasonal variation) and were fed naturally occurring algal communities cultivated in an earthen ponds using natural seawater, with a 12 h light:12 h dark photoperiod, mimicking field-like conditions, for two years. In June 2024, mussels from two families were further selected, to investigate whether variation among selectively-bred families influences the effectiveness of the pre-treatments in mitigating live transport stress (see below and Fig. 1). These two families were selected due to their sufficient number for experimentation and due to contrasting differences in their thermal tolerances. These included a less heat-tolerant family (i.e., Family C, hereafter FamC) and a more heat-tolerant family (i.e., Family F, hereafter FamF), identified based on thermal tolerance results from Delorme et al. (2024b). Using these two families enabled assessment of pre-treatment effectiveness across different selectively-bred mussels. Ethical approval was not required for this study, as mussels are invertebrates and are not covered under the Animal Welfare Act issues by the New Zealand government and therefore not required by the university’s animal ethics committee.

### Experimental procedures

#### Post-harvest and pre-transport treatment conditions

For logistical reasons, the experimental work was divided between two identical rounds, each with 12 mussels randomly picked up from each of the selected families to avoid any potential effects due to size variation (i.e., FamC and FamF). Across both rounds, this resulted in a total of 96 mussels, including control and treatment groups (see end of Sect. 2.2.2). Experimental mussels were carefully separated from each other by cutting their byssal threads and transferred to the adjacent laboratory for experimentation. Mussels were placed on aluminium trays at room temperature in air (16 °C) for two hours to simulate post-harvest process (Cheng et al. 2025b). All mussels were subsequently treated by submersing the mussels in water containing 40 g of MgCl_2_ L^− 1^ maintained at room temperature (16.4 ± 0.2 °C) for two hours with aeration, which demonstrated that this treatment effectively reduce mussel metabolic activity while maintaining mussel viability during live transport simulation (Cheng et al., 2026). Temperatures were maintained using a water bath connected to chillers (Hailea HC-500 A, China) and a circulation pump, with temperature monitored every ten minutes by temperature loggers (EnvLogger T2.4, Electriblue, Portugal). The MgCl_2_ solution was prepared by dissolving MgCl_2_·6H_2_O in a mixture of seawater (salinity ~ 35) and freshwater (3:7 v/v), under continuous stirring until complete dissolution and full homogeneity were achieved before use, to account for the effect of the added MgCl_2_ on total osmolality. The volume prepared ensured that each mussel was exposed to 0.5 L of solution (after Cheng et al. 2025b). The mussels were left in the treatment for two hours.

#### Live transport simulation and subsequent recovery, and tissue sampling

After MgCl_2_ pre-treatment, the mussels were placed in aluminium trays (i.e., one tray per family) within a large container connected to a water bath and chiller (Hailea HC-500 A, China) to maintain air temperature at 8.1 ± 0.5 °C and relative humidity above 90% for three days to simulate long-distance live transport (Barrento et al., 2013; Cheng et al., 2026). All mussels were positioned with the right valves facing downwards to minimise potential effects of shell orientation. Air temperature and humidity were monitored using iButtons (DS1923-F5 Hygrochron, iButton^®^, USA). The transport temperature was selected based on live shellfish transportation guidelines from the Ministry for Primary Industries (MPI), New Zealand (MPI, 2017).

After the three-day live transport simulation (i.e., the first time point; TP1), four mussels from each of the two selected families were sampled for gill tissues. Mussels were shucked using a blunt knife from the posterior end. Left- and right-side gill lamellae were dissected and placed separately into labelled 2 mL cryogenic tubes, immediately snap-frozen in liquid nitrogen, and stored at −80 °C until analysis. At the same time, a separate group of four mussels from each family was sampled directly from the holding tanks to serve as controls.

Following the live transport simulation, the remaining mussels were transferred to four 150 L tanks with flow-through filtered seawater (1 μm, salinity 35) maintained at 12.4 ± 0.9 °C. Mussels were fed *ad libitum* with a 1:1 mixture of *Tisochrysis lutea* and *Chaetoceros muelleri* (~ 8 µg Chl *a* L^− 1^) for five days, during which mortality was monitored. A previous study (Cheng et al., 2026) indicated that metabolite profiles of mussels can recover within one day post-transport (i.e., metabolic profiles retuned to baseline levels). A 5-day recovery period was, therefore, chosen to ensure that any metabolic changes from transport to full recovery could be captured.

In the meantime, eight control mussels from each family were transferred from the holding tanks directly to the laboratory recovery tanks to account for potential effects of changing food environments on mussel metabolic profiles. These mussels served as the control group and were exposed to the same food transition as the experimental group (i.e., the control mussels were not subjected to either the MgCl_2_ treatment or the live transport simulation). This approach ensured that any metabolic changes due to the shift in food environment were equally represented in both control and treated mussels, allowing for a more accurate interpretation of treatment effects. Gill tissues were sampled from four mussels per family after 1 day and 5 days recovery (i.e., TP2 and TP3) from live transport. Similarly, gill tissues from four control mussels per family were collected at the corresponding time points (i.e., TP2 and TP3). No mortality of mussels from these families was observed throughout the experiment. To capture potential inter-familial physiological variability in stress responses, detailed metabolomic profiling was carried out. After two experimental rounds, eight experimental mussels and eight control mussels were sampled per time point per family (i.e., ∑*n* = 2 groups × 3 time points × 8 mussels × 2 families = 96) for metabolomic analysis.

### Metabolic profiling

Metabolic profiling of mussel gill tissues was conducted at the Mass Spectrometry Centre, University of Auckland (UoA), using gas chromatography-mass spectrometry (GC-MS) following the protocol developed by Smart et al. (2010) and further detailed in Cheng et al. (2025b). Frozen tissues were freeze-dried overnight and directly homogenised in sample tubes using a stainless steel grinding rod until a fine powder was obtained. Approximately 10 mg of each homogenised sample was transferred to a 1.5 mL microcentrifuge tube and mixed with 20 µL of 10 mM d_4_-Alanine as an internal standard. Metabolites were extracted in two steps. First, 500 µL of cold 50% methanol-water was added, and samples were centrifuged at −9 °C and 1,150 rcf for five minutes. The supernatant was collected into a 2 mL Eppendorf tube. A second extraction was then performed using 500 µL of 80% cold methanol-water, following the same procedure. Supernatants from both extractions were combined and dried using a SpeedVac concentrator (Savant^™^ SC250EXP, Thermo Scientific). Dried extracts were stored at −80 °C until derivatisation.

For derivatisation, dried metabolites were reconstituted in 400 µL of 1 M sodium hydroxide, followed by the addition of 334 µL methanol and 68 µL pyridine. Methyl chloroformate (MCF) was added in two steps: 40 µL with vortexing for 30 s, followed by another 40 µL and an additional 30 s of vortexing. The derivatised products were extracted using 400 µL chloroform followed by addition of 800 µL of 50 mM sodium bicarbonate. After centrifugation at 6 °C and 845 rcf for five minutes, the upper aqueous layer was removed. A small amount of sodium sulfate powder was added to the chloroform phase to remove residual moisture. The chloroform phase was then transferred to glass micro-inserts and placed into GC vials for analysis.

Derivatised samples were analysed using a gas chromatograph (GC7890B, Agilent Technologies, USA) connected to a quadrupole mass spectrometer (MSD5977A, Agilent Technologies, USA), operating at 70 eV electron ionisation. A 1 µL aliquot was injected in splitless mode at 290 °C, with helium as the carrier gas at a constant flow of 1 mL min^−1^ through a ZB-1701 (14% cyanopropylphenyl and 86% dimethylpolysiloxane as stationary phase) capillary column (30 m × 250 μm ID × 0.15 μm film thickness). The GC oven temperature programme began at 45 °C (held for two minutes), followed by a ramp to 180 °C at 9 °C min^−1^ (held for five minutes). Then, the temperature was ramped at a constant rate of 40 °C min^−1^ to 220 °C (held for five minutes), subsequently to 240 °C (held for 11.5 min) and finally to 280 °C, which was maintained for ten minutes. Ion source and quadrupole temperatures were 230 °C and 150 °C, respectively. The mass spectrometer was operated in scan mode (38–550 m/z, four scans per second) with a detection threshold of 50 ion counts. Quality control (QC) was ensured by including both non-derivatised alkane standard mixtures and derivatised quality control samples. The QC samples, including a mixture of amino and organic acids, as well as blanks and pooled tissue extracts, were co-derivatised with 20 µL of 10 mM d_4_-Alanine. These QC samples were injected at the start of the GC-MS run and then intermittently after every 17 samples. The remaining samples were analysed in a randomised order.

Raw mass spectrometry data were processed using AMDIS software (NIST, USA) and the in-house R package “MassOmics” (https://github.com/MASHUOA/MassOmics), developed by the University of Auckland (UoA), for GC-MS metabolite identification following MCF derivatisation (Smart et al., 2010). Metabolite identification and peak integration were conducted using in-house mass spectral libraries for MCF-derivatised standards developed by UoA. Identified compounds were manually verified, and only those with a match score of ≥ 75%, based on both MS spectra and retention time, were retained for peak integration to determine metabolite abundance (i.e., peak area). Integrated peaks were batch-filtered using the “MassOmics” R package, with additional manual checks in ChemStation (Agilent Technologies, USA) and AMDIS to remove potential laboratory contaminants. Duplicate and abnormal entries were excluded based on detection frequency (i.e., how often a compound appeared across all samples), reference ion, retention time, and peak area. The cleaned dataset was normalised to the internal standard (d_4_-Alanine), corrected using blank samples to account for variation introduced during sample handling, and finally standardised by dry sample biomass.

### Statistical analysis

#### Global metabolic changes after live transport and recovery

Statistical analyses were performed using R (version 4.5.1; R Core Team, 2025). Before conducting any analyses, the metabolomic data were normalised using autoscaling (i.e., each metabolite concentration was mean-centred and divided by its standard deviation). Permutational multivariate analysis of variance (PERMANOVA; Anderson, 2001) was used to assess the effects of fixed factors including family, sampling time point, pre-treatment, and their interactions on metabolic profiles. Prior to PERMANOVA, homogeneity of multivariate dispersion (i.e., spread of data within each group) was tested to check if group dispersions are equal, ensuring that differences detected by PERMANOVA are due to real group differences, rather than unequal within-group dispersion (Anderson, 2006). To account for potential variability between experimental rounds as a random effect, permutations were restricted within each round. This analysis was performed using an R package “vegan” (Oksanen et al., 2024) with 999 permutations. Once there was a significant interaction between time point (TP) and pre-treatments (Trt) (i.e., TP × Trt < 0.05; Table 1), principal coordinate analysis (PCoA) was applied to pooled family data to visualise overall differences in metabolic profiles between control and MgCl_2_ pre-treated mussels across time points: after a 3-day live transport simulation (TP1), after a subsequent 1 day seawater recovery (TP2), and after 5 day seawater recovery (TP3). To determine whether significant differences existed between treatment groups at each time point, PERMANOVA was conducted separately at each time point to compare the metabolic profiles of control and MgCl_2_ pre-treated mussels. This analysis was to assess the overall effect of MgCl_2_ treatment across all mussels, regardless of family, as an initial overview.

**Table 1:** PERMANOVA results comparing variation in gill metabolic profiles of mussels based on family (Fam), pre-treatment (Trt: control and MgCl_2_), time point (TP: immediately after 72-hour aerial exposure at 9 °C for live transport simulation, after 1 day recovery in seawater, and after 5 day recovery in seawater), and their interactions

Source	df	MS	Pseudo-F	*p* value
Fam	1	372.10	7.098	**0.001**
TP	2	455.75	8.694	**0.001**
Trt	1	467.70	8.922	**0.001**
TP × Trt	2	185.60	3.540	**0.001**
Fam × TP × Trt	5	73.58	1.404	**0.044**
Residual	82	52.42		

The analysis was performed with α: 0.05. Significant *p*-values are shown in bold

#### Time-dependent metabolic responses by pre-treatment and family

Following the detection of a significant interaction between family (Fam), time point (TP), and pre-treatments (Trt) in the overall PERMANOVA (i.e., Fam × TP × Trt < 0.05; Table 1), we conducted a subsequent exploratory analysis to visualise and compare overall metabolic patterns. Both PCoA and PERMANOVA were subsequently performed separately for each mussel family (FamC and FamF) at each sampling time point (TP1, TP2 and TP3) to examine differences in metabolic profiles between control and MgCl_2_ pre-treated mussels. This analysis was intended to support visual interpretation of group-level separation in multivariate space rather than to test individual metabolite-level significance. To further outline the differences in metabolic profiles between the pre-treatments (fixed factor) at different time points (fixed factor), a two-way ANOVA was individually deployed on each metabolite using linear mixed effects model (LMM), which is robust despite assumption violations (Schielzeth et al., 2020), from an R package “glmmTMB” (Brooks et al., 2017) while considering the effects of different trials (i.e., random factor). Metabolites that were significantly affected by pre-treatments and time points (*p* < 0.05) were selected for each family. Concentrations of the selected metabolites were visualised using heatmaps with hierarchical clustering, generated with the R package “pheatmap” (Kolde, 2019). Dendrograms produced through hierarchical clustering were integrated into the heatmaps to illustrate similarities among metabolites and to display their concentration patterns across different pre-treatments and time points.

#### Temporal changes in metabolic pathways by treatment and family

To reveal related metabolic pathways affected during live transport simulation and recovery, pathway analysis was conducted. Before that, metabolites, which were significantly different between MgCl_2_ pre-treated mussels and the corresponding time- and family-matched control group (i.e., mussels not subjected to MgCl_2_ treatment or live transport simulation; see Sect. 2.2.2), were identified at each time point for each family with one-way analysis of variance (ANOVA) using LMM from R package “glmmTMB” while considering the effects of different rounds (i.e., random factor). To ensure a more stringent criteria for extracting metabolites affected for downstream pathway analysis, *p* values were adjusted using the False Discovery Rate (FDR) approach according to the Benjamini-Hochberg procedure. Metabolites with FDR-adjusted *p* (*p*_adj_) values below 0.05 were considered statistically significant.

Fold changes (FC) of statistically significant metabolites were calculated using non-normalised data by dividing the mean concentration in the control group by that in the pre-treatment group (MgCl_2_). FC were then log_2_-transformed (log_2_FC), and metabolites with absolute log_2_FC > 0.58, indicating at least a 1.5-fold increase or decrease to screen for biological relevance (Forsberg et al., 2018), were further selected. Partial least squares discriminant analysis (PLS-DA) was conducted using the R package “mixOmics” (Rohart et al., 2017) to calculate variable importance in projection (VIP) scores. Metabolites with VIP scores > 1, indicating a strong contribution to group separation between control and MgCl_2_ pre-treatment groups at each time point for each family, were considered relevant. To increase confidence in identifying true metabolic responses, metabolites that met all three criteria, including statistical significance (*p*_adj_ < 0.05), absolute log_2_FC > 0.58, and VIP > 1, were defined as differentially expressed metabolites (DEMs). This integrative approach ensures that selected metabolites are not only statistically robust but also important in showing real biological differences between the groups. These DEMs were subsequently used for pathway analysis (*sensu* Xia et al., 2010).

Pathway analysis was conducted using MetaboAnalyst 6.0 (Pang et al., 2024; https://www.metaboanalyst.ca/) based on DEMs identified between control and post-harvest groups at each time point for each mussel family. Metabolites were mapped to metabolic pathways of *Schistosoma mansoni* (a flatworm species with a closer phylogenetic relationship to molluscs than other species with bioinformatic data available on online database; Adoutte et al., 2000) using the Kyoto Encyclopedia of Genes and Genomes (KEGG) database (Kanehisa & Goto, 2000). This organism was selected because commonly used KEGG model species are often distantly related to molluscs, which may reduce the reliability of pathway mapping even when metabolites are conserved. *S. mansoni* was, therefore, used as a closer phylogenetic proxy to improve the biological relevance of pathway inference, while acknowledging limitations in pathway annotation for non-model organisms. All annotated metabolites detected in this study (Table S1) were used as the reference compound set (Wieder et al., 2021). Pathways involving more than two annotated metabolites (i.e., DEMs) that matched the KEGG database with *p* value < 0.05, FDR < 0.1 and impact score > 0.1 were regarded as significantly enriched pathways of interest.

## Results

### Global metabolic changes after live transport simulation and recovery

**Fig. 2:**
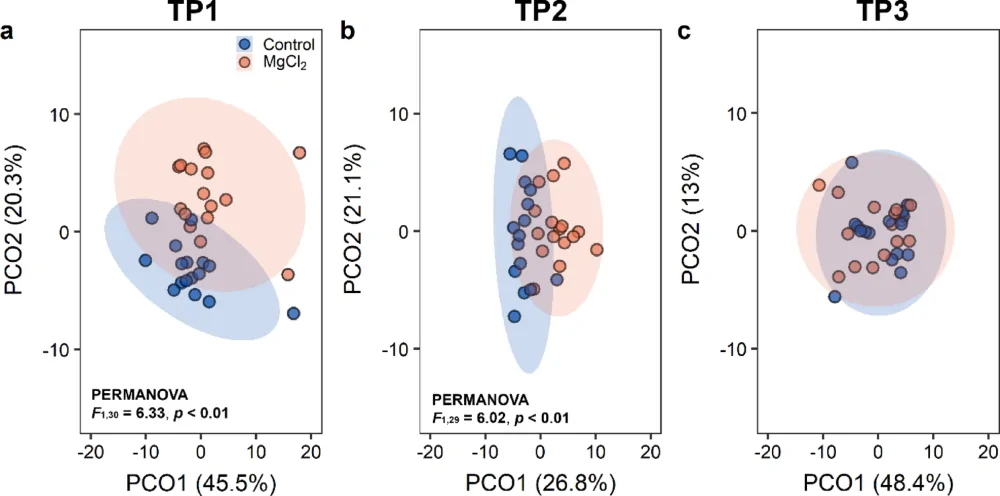
Principal Coordinate Analysis (PCoA) plots showing differences in gill metabolic profiles of mussels (all families pooled) between control (i.e., mussels directly sampled from the holding tanks and were not subjected to either the MgCl_2_ treatment or the live transport simulation) and MgCl_2_ (i.e., mussels experienced MgCl_2_ pre-treatment) groups across different time points (TP). Mussels were sampled immediately after 72-hour aerial exposure at 9 °C for live transport simulation (TP1, **a**), after 1-day recovery in seawater (TP2, **b**) and after 5 days of recovery (TP3, **c**). Shaded areas represent 95% confidence ellipses

**Fig. 3:**
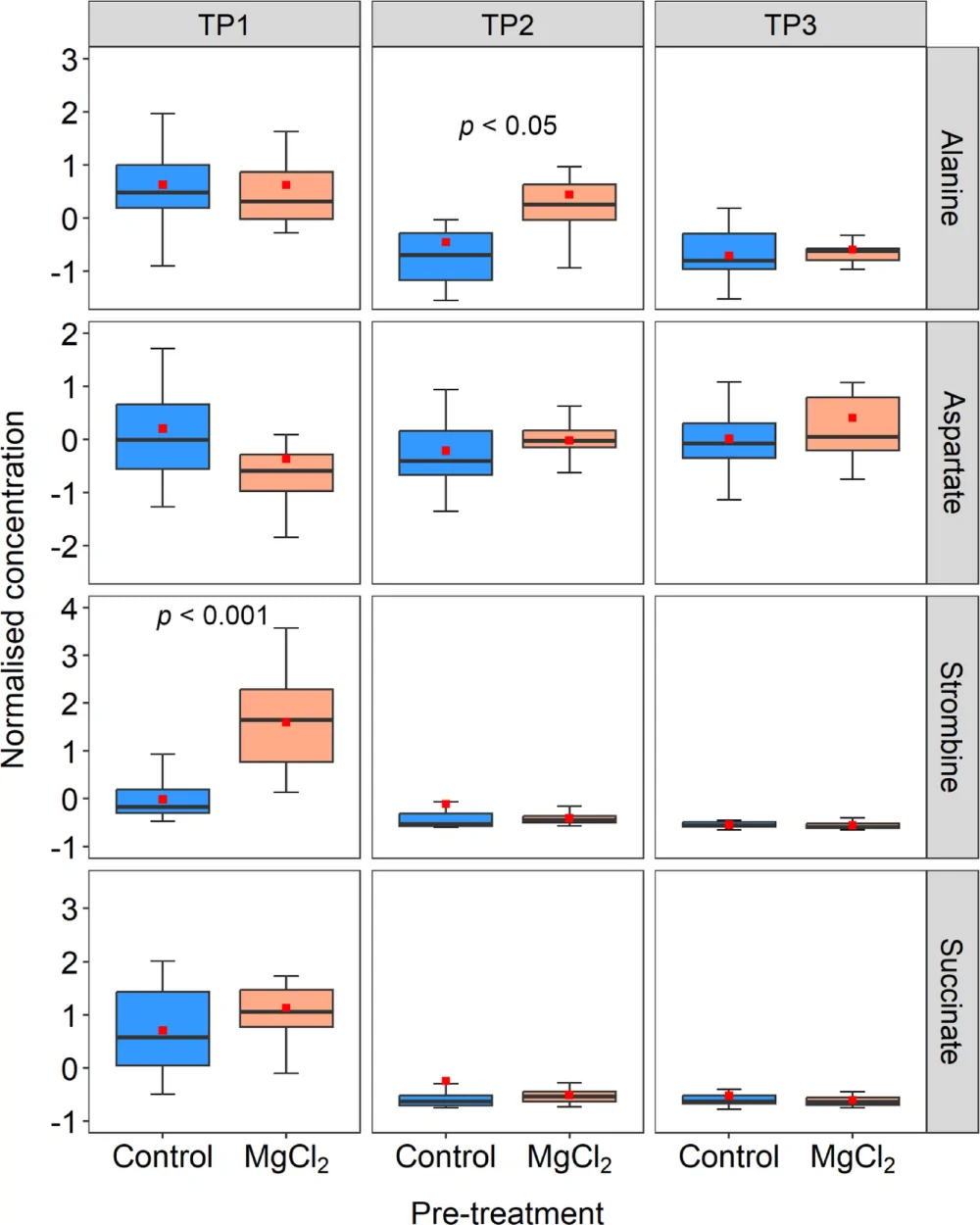
Boxplots illustrating the concentrations of key biomarkers associated with anaerobic energy production of mussels during the recovery period. The mussels were subjected to MgCl_2_ pre-treatments following a 72-hour aerial exposure at 9 °C, simulating live transport, while control mussels were not subjected to either the MgCl_2_ treatment or the live transport simulation. Red dots indicate the mean values. Samples were collected after live transport simulation (TP1), 1 day (TP2) and 5 days (TP3) after recovery. Graphs without significance annotations indicate no significant differences between the two groups

Metabolic profiles with pooled mussel family data were significantly influenced by both pre-treatment and time point (PERMANOVA, Time point (TP) × pre-treatment (Trt), *p* < 0.01; Table 1). A significant difference was observed between control and MgCl_2_ pre-treated mussels at TP1 (immediately after 3 day live transport simulation, *p* < 0.01; Fig. 2a) and TP2 (after 1 day recovery, *p* < 0.01; Fig. 2b), with metabolomes returning to levels comparable to those of control mussels at TP3 (i.e., after 5 days of recovery; Fig. 2c). Generally, MgCl_2_ dampened, but not completely eliminated, the transport-stress, as reflected in slight differences in anaerobic biomarkers after live transport simulation and early recovery phase (Fig. 3).

### Family- specific metabolic responses to live transport and recovery

**Fig. 4:**
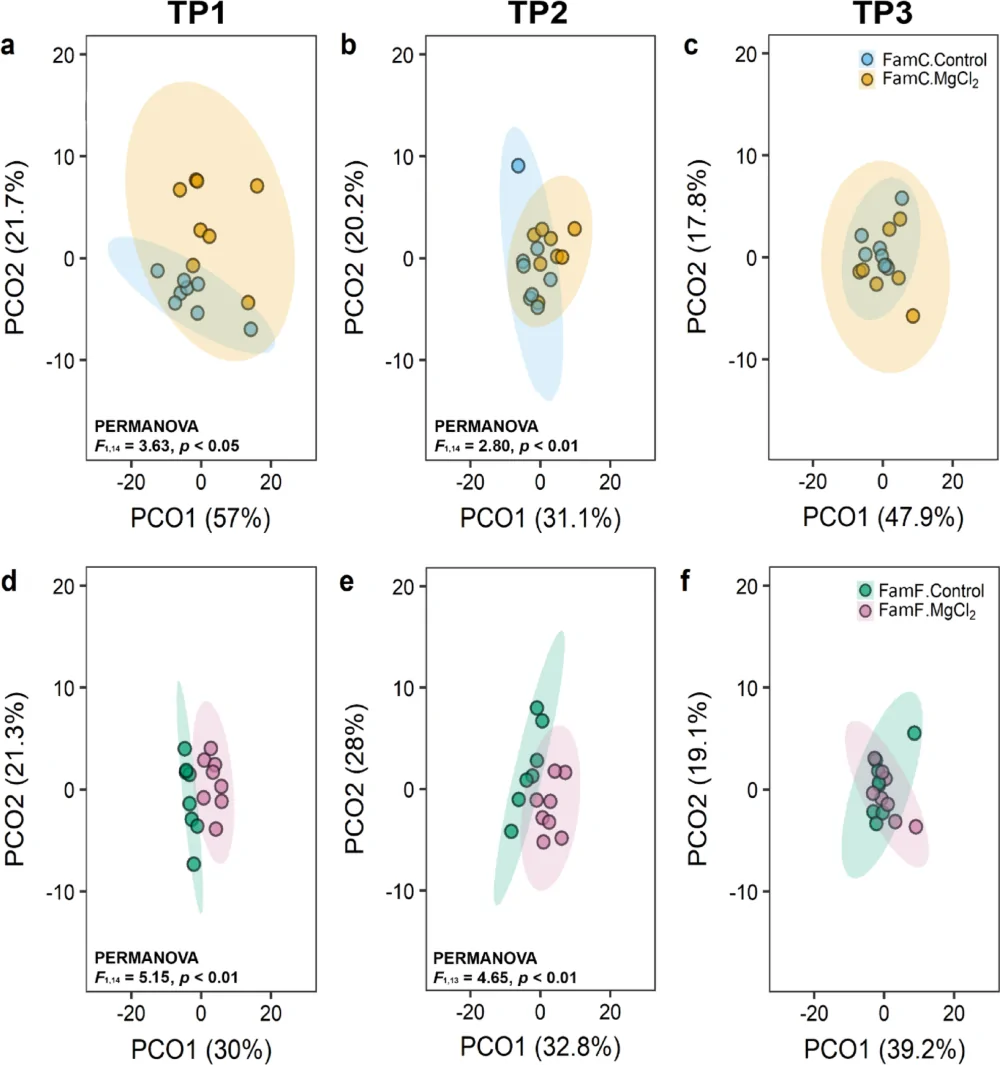
Principal Coordinate Analysis (PCoA) plots showing differences in gill metabolic profiles of mussels between control (i.e., mussels directly sampled from the holding tanks and were not subjected to either the MgCl_2_ treatment or the live transport simulation) and MgCl_2_ (i.e., mussels experienced MgCl_2_ pre-treatment) groups from a less heat-tolerant family (FamC, upper panel) and a more heat-tolerant family (FamF, lower panel) across different time points (TP). Mussels were sampled immediately after 72-hour aerial exposure at 9 °C for live transport simulation (TP1: **a**, **d**), after 1-day recovery in seawater (TP2: **b**, **e**) and after 5-day recovery (TP3: **c**, **f**). Shaded areas represent 95% confidence ellipses

A significant three-way interaction was detected in the metabolic profiles of mussels, indicating that family (Fam), time point (TP), and pre-treatment (Trt) interacted to influence metabolic profiles (Fam × TP × Trt, *p* < 0.05; Table 1). At TP1, significant differences in metabolic profiles were observed between control and MgCl_2_ pre-treated mussels from both FamC and FamF (*p* < 0.05; Fig. 4a, d). These differences persisted after one day of recovery (TP2, *p* < 0.01; Fig. 4b, e). By TP3 (after 5-day recovery), the metabolomes returned to levels comparable to those of the control mussels (Fig. 4c, f).

**Fig. 5:**
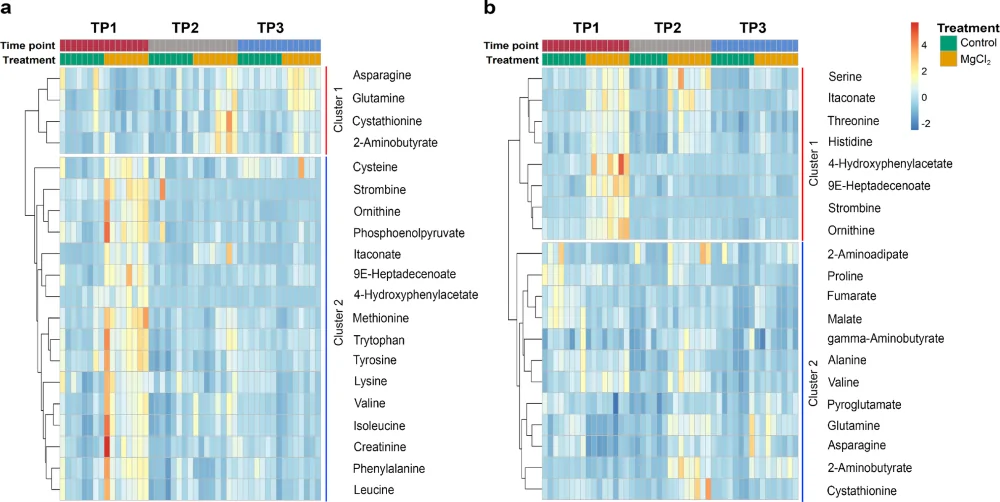
Heatmaps showing the top 20 significantly different metabolites (*p* < 0.05) of mussels between control (i.e., mussels directly sampled from the holding tanks and were not subjected to either the MgCl_2_ treatment or the live transport simulation) and MgCl_2_ (i.e., mussels experienced MgCl_2_ pre-treatment) groups at three sampling time points: TP1 (immediately after 72-hour aerial exposure at 9 °C for live transport simulation), TP2 (after 1-day recovery) and TP3 (after 5-day recovery in seawater) from a less heat-tolerant family (FamC, left panel **a**) and a more heat-tolerant family (FamF, right panel **b**)

There were, respectively, 28 and 25 metabolites in FamC and FamF that were significantly different between control and MgCl_2_ pre-treated mussels across different time points (one-way ANOVA *p* < 0.05, Table S2). Heatmaps illustrate the relative abundance of the top 20 metabolites (ranked according to *p*-values in ascending order from smallest to largest) that significantly differed between control and MgCl_2_ pre-treated mussels across different time points for FamC and FamF (Fig. 5), forming two distinct clusters. In FamC mussels, Cluster 1 metabolites, putatively associated with cellular homeostasis and stress-related processes, showed a moderate increase across sampling time points relative to the control group. In contrast, Cluster 2 metabolites linked to energy metabolism, antioxidant defence and neurotransmitter biosynthesis, were higher after the 3-day live transport simulation compared to other time points and returned to levels similar to control mussels during the recovery phase (Fig. 5a). In FamF mussels, Cluster 1 metabolites associated with cellular stress-related processes were elevated after live transport simulation but were comparable to control levels after 1-day recovery. Cluster 2 metabolites associated with energy and amino acid metabolism, osmoregulation and cellular stress-related processes were lower following transport and showed a slight increase during the recovery period (Fig. 5b).

### Family-specific metabolic pathways after live transport and recovery

**Fig. 6:**
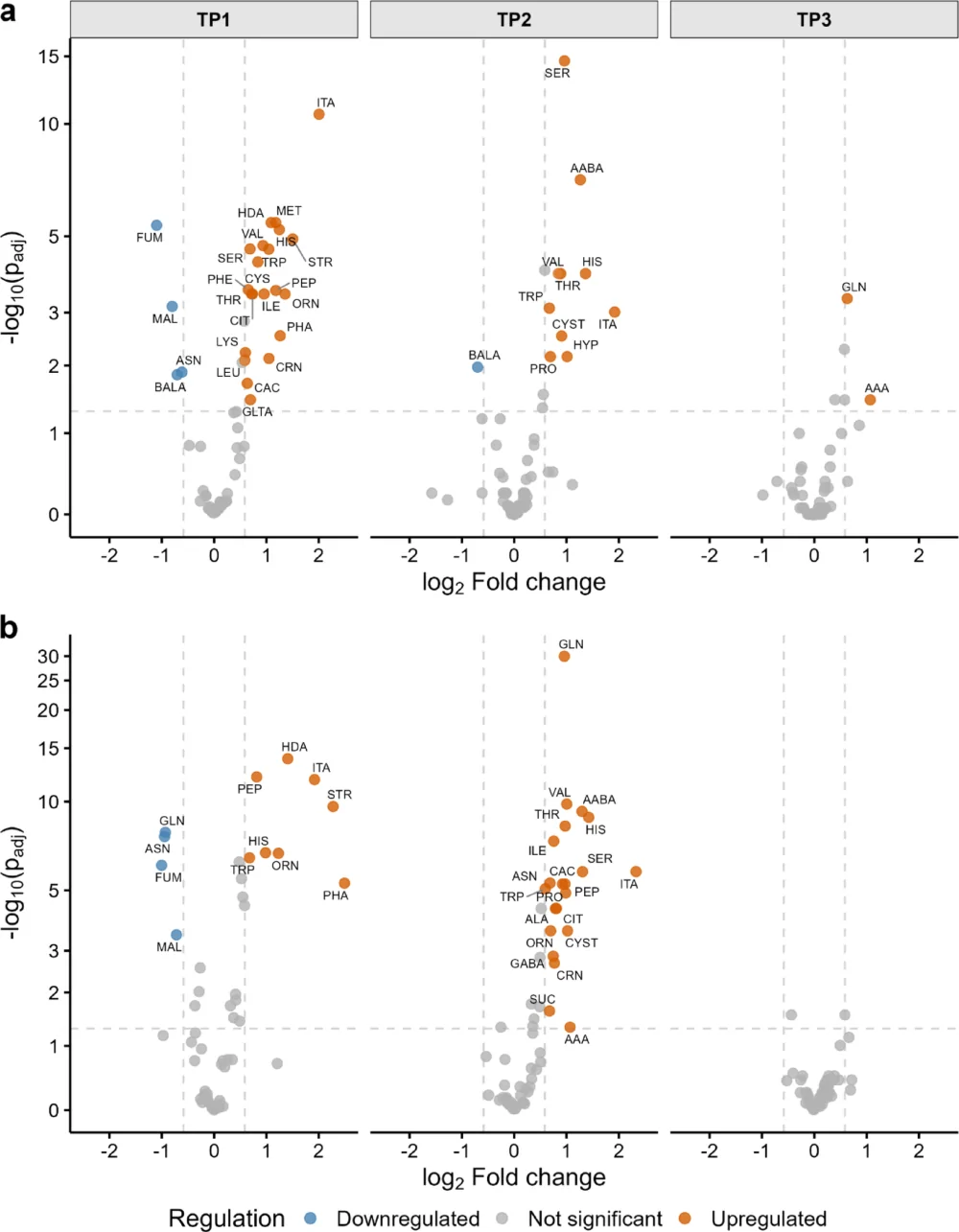
Volcano plot showing metabolite expression at different time points: TP1 (immediately after 72-hour aerial exposure at 9 °C for live transport simulation), TP2 (after 1-day recovery) and TP3 (after 5-day recovery in seawater) from (**a**) a less heat-tolerant family (FamC) and (**b**) a more heat-tolerant family (FamF). Each point represents a metabolite, with the y-axis showing –log_10_(p-value) and the x-axis indicating log_2_ fold change (FC). FC was calculated by comparing MgCl_2_ pre-treated mussels with the corresponding time- and family-matched control group. Significantly upregulated (log_2_FC > 0.58; *p*_adj_ < 0.05) and downregulated (log_2_FC < −0.58; *p*_adj_ < 0.05) metabolites are shown in red and blue respectively. Non-significant metabolites are shown in grey. Dashed vertical lines represent the fold change threshold (log_2_FC = ± 0.58, equivalent to FC = 1.5), and the horizontal line marks the significance threshold (*p*_adj_ = 0.05). The y-axis uses a non-linear (compressed) scale to visually accommodate high p-values. See Table 2 for the full names of the metabolites

Metabolomic analysis of gill tissues from FamC and FamF mussels revealed distinct differences in metabolic profiles between control and MgCl_2_ pre-treated groups across sampling time points. These differences were highlighted in the PLS-DA plots, which showed clear group separation driven by metabolites with high VIP scores (Fig. S1). The number of DEM (one-way ANOVA, *p*_*adj*_ < 0.05, |log_2_FC| > 0.58, VIP scores > 1) decreased with sampling time point from 25 (4 downregulated and 21 upregulated, Table 2; Fig. 6a) at TP1 to 2 at TP3 (all upregulated) for FamC mussels (Table S3). While DEM increased from 12 (4 downregulated and 8 upregulated, Table 2; Fig. 6b) to 21 (all upregulated) from TP1 to TP2 and there is no DEM at TP3 for FamF mussels (Table S4).

**Table 2:** Differentially expressed metabolites identified in gill tissues of the less heat-tolerant (FamC) and more heat-tolerant (FamF) mussel families at three time points: (a) TP1, immediately after 72-hour aerial exposure at 9 °C for live transport simulation; (b) TP2, after 1-day recovery in seawater; and (c) TP3, after 5-day recovery in seawater

Category	Metabolite	FamC			FamF		
		TP1	TP2	TP3	TP1	TP2	TP3
Amino acid	Alanine (ALA)					**↑**	
Amino acid	Asparagine (ASN)	**↓**			**↓**	**↑**	
Amino acid	beta-Alanine (BALA)	**↓**	**↓**				
Amino acid	Cysteine (CYS)	**↑**					
Amino acid	Glutamine (GLN)			**↑**	**↓**	**↑**	
Amino acid	Histidine (HIS)	**↑**	**↑**		**↑**	**↑**	
Amino acid	Isoleucine (ILE)	**↑**				**↑**	
Amino acid	Leucine (LEU)	**↑**					
Amino acid	Lysine (LYS)	**↑**					
Amino acid	Methionine (MET)	**↑**					
Amino acid	Ornithine (ORN)	**↑**			**↑**	**↑**	
Amino acid	Phenylalanine (PHE)	**↑**					
Amino acid	Proline (PRO)		**↑**			**↑**	
Amino acid	Serine (SER)	**↑**	**↑**			**↑**	
Amino acid	Threonine (THR)	**↑**	**↑**			**↑**	
Amino acid	Tryptophan (TRP)	**↑**	**↑**		**↑**	**↑**	
Amino acid	Valine (VAL)	**↑**	**↑**			**↑**	
NAA	2-Aminobutyrate (AABA)		**↑**			**↑**	
NAA	gamma-Aminobutyrate (GABA)					**↑**	
NAA	Hydroxyproline (HYP)		**↑**				
AAD	4-Hydroxyphenylacetate (PHA)	**↑**			**↑**		
AAMI	2-Aminoadipate (AAA)			**↑**		**↑**	
AAMI	Creatinine (CRN)	**↑**				**↑**	
AAMI	Cystathionine (CYST)		**↑**			**↑**	
Imino acid	Strombine (STR)	**↑**			**↑**		
Fatty acid	9E-Heptadecenoate (HDA)	**↑**			**↑**		
Organic acid	*cis*-Aconitate (CAC)	**↑**				**↑**	
Organic acid	Citrate (CIT)	**↑**				**↑**	
Organic acid	Fumarate (FUM)	**↓**			**↓**		
Organic acid	Glutarate (GLTA)	**↑**					
Organic acid	Itaconate (ITA)	**↑**	**↑**		**↑**	**↑**	
Organic acid	Malate (MAL)	**↓**			**↓**		
Organic acid	Phosphoenolpyruvate (PEP)	**↑**			**↑**	**↑**	
Organic acid	Succinate (SUC)					**↑**	

These metabolites were used for pathway analysis. *AAD* Amino Acid Derivative, *AAMI* Amino Acid Metabolic Intermediate, *NAA* Non-standard Amino Acid. No differentially expressed metabolites were identified in FamF mussels at TP3. The symbols **↓** and **↑** represent downregulated and upregulated metabolites, respectively

For FamC mussels, the number of identified metabolic pathways decreased from 19 at TP1 to 8 at TP2 and 5 at TP3. Of these, 10 pathways at TP1, 4 at TP2, and 2 at TP3 were of particular interest, mainly related to energy metabolism, amino acid metabolism, and cellular stress responses, based on significantly altered metabolites with *p*_adj_ < 0.05, FDR < 0.1, and impact scores > 0.1 (Table 3, S5). For FamF mussels, 11 pathways were identified at TP1, and 5 were associated with energy and amino acid metabolism. At TP2, 16 pathways were detected, and 10, involved in energy metabolism, amino acid metabolism, and cellular stress responses, were of interest (Table 4, S6). By comparison, FamC mussels had more pathways enriched after live transport simulation (TP1), whereas FamF mussels had more pathways enriched after 1-day recovery (TP2).

**Table 3:** List of significantly enriched metabolic pathways of interest in mussels from a less heat-tolerant family (FamC) sampled at different time points: (a) TP1, immediately after 72-hour aerial exposure at 9 °C for live transport simulation; (b) TP2, after 1-day recovery in seawater; and (c) TP3, after 5-day recovery in seawater

Pathway	Detected enriched metabolites	Hits	−log(*p*)	FDR	Impact
(a) TP1					
Citrate cycle (CC)	Malate, *cis*-aconitate, citrate, fumarate, phosphoenolpyruvate	5 [6]	5.818	< 0.001	0.214
Cysteine and methionine metabolism (CMM)	Serine, methionine, cysteine	3 [6]	4.463	< 0.001	0.387
One carbon pool by folate (OCP)	Methionine, serine, cysteine	3 [6]	4.463	< 0.001	0.222
Glyoxylate and dicarboxylate metabolism (GDM)	*cis*-aconitate, malate, citrate, serine	4 [7]	4.023	< 0.001	0.537
Glycine, serine and threonine metabolism (GST)	Serine, threonine, cysteine	3 [5]	3.413	0.001	0.326
Tryptophan metabolism (TPM)	Tryptophan	1 [1]	3.188	0.001	0.235
Phenylalanine metabolism (PM)	Phenylalanine	1 [1]	2.722	0.002	1.000
Glycolysis/gluconeogenesis (GG)	Phosphoenolpyruvate	1 [1]	2.615	0.002	0.101
Arginine and proline metabolism (APM)	Ornithine	1 [4]	2.612	0.002	0.148
Arginine biosynthesis (AB)	Ornithine	1 [3]	2.612	0.002	0.143
(b) TP2					
Glycine, serine and threonine metabolism (GST)	Serine, cystathionine, threonine	3 [5]	2.7882	0.004	0.326
One carbon pool by folate (OCP)	Serine, cystathionine	2 [6]	2.6185	0.004	0.398
Cysteine and methionine metabolism (CMM)	Cystathionine, serine	2 [6]	2.6185	0.004	0.366
Tryptophan metabolism (TPM)	Tryptophan	1 [1]	1.5031	0.031	0.235
(c) TP3					
Arginine biosynthesis (AB)	Glutamine	1 [3]	2.977	0.001	0.214
Alanine, aspartate and glutamate metabolism (AAG)	Glutamine	1 [6]	2.977	0.001	0.196

The abbreviation for each metabolic pathway is in parentheses. Numbers in the square brackets represent the total number of compounds involved in the metabolic pathways detected in this study

**Table 4:** List of significantly enriched metabolic pathways of interest in mussels from a more heat-tolerant family (FamF) sampled at different time points (a) TP1, immediately after 72-hour aerial exposure at 9 °C for live transport simulation; and (b) TP2, after 1-day recovery in seawater

Pathway	Detected enriched metabolites	Hits	−log(*p*)	FDR	Impact
(a) TP1					
Arginine biosynthesis (AB)	Glutamine, ornithine	2 [3]	5.560	< 0.001	0.357
Alanine, aspartate and glutamate metabolism (AAG)	Glutamine, fumarate	2 [6]	5.042	< 0.001	0.196
Arginine and proline metabolism (APM)	Ornithine	1 [4]	3.660	< 0.001	0.148
Glycolysis/gluconeogenesis (GG)	Phosphoenolpyruvate	1 [1]	3.533	< 0.001	0.101
Tryptophan metabolism (TPM)	Tryptophan	1 [1]	2.301	0.005	0.235
(b) TP2					
Arginine biosynthesis (AB)	Glutamine, ornithine	2 [3]	7.212	< 0.001	0.357
Alanine, aspartate and glutamate metabolism (AAG)	Alanine, glutamine, succinate	3 [6]	7.086	< 0.001	0.196
Glyoxylate and dicarboxylate metabolism (GDM)	*cis*-aconitate, citrate, serine, glutamine	4 [7]	4.925	< 0.001	0.537
Glycine, serine and threonine metabolism (GST)	Serine, cystathionine, threonine	3 [5]	4.553	< 0.001	0.326
One carbon pool by folate (OCP)	Serine, cystathionine	2 [6]	4.301	< 0.001	0.398
Cysteine and methionine metabolism (CMM)	Cystathionine, serine	2 [6]	4.301	< 0.001	0.366
Arginine and proline metabolism (APM)	gamma-aminobutyrate, ornithine, proline	3 [4]	3.843	< 0.001	0.262
Citrate cycle (CC)	Succinate, *cis*-aconitate, citrate, phosphoenolpyruvate	4 [6]	3.294	< 0.001	0.173
Tryptophan metabolism (TPM)	Tryptophan	1 [1]	3.216	< 0.001	0.235
Glycolysis/gluconeogenesis (GG)	Phosphoenolpyruvate	1 [1]	1.469	0.034	0.101

There are no differentially expressed metabolites selected in TP3 (after 5-day recovery in seawater) for pathway analysis. The abbreviation for each metabolic pathway is in parentheses. Numbers in the square brackets represent the total number of compounds involved in the metabolic pathways detected in this study

## Discussion

Two distinct families of mussel, *Perna canaliculus*, were chosen from a selective breeding programme and tested for their ability to cope with a simulated live transport. Metabolic profiles, however, varied between control and MgCl_2_ pre-treated mussels across sampling time points, and these differences were also influenced by family, with distinct patterns observed between two families known to have contrasting acute heat tolerance (Delorme at al., 2024b). FamC mussels showed an earlier and stronger metabolic response, whereas FamF displayed a delayed but more pronounced response at TP2, suggesting slower physiological adjustment to transport stress in the more thermotolerant family.

### MgCl_2_ effects during live transport and recovery

Live transport exposes shellfish to fluctuating conditions such as temperature changes, desiccation, mechanical disturbance and accumulation of metabolic waste, while prolonged aerial exposure can limit oxygen availability and potentially induce hypoxia (Mohamed & Devaraj, 1997; Fotedar & Evans, 2011). Under such stress, bivalves may shift from aerobic to anaerobic metabolism, leading to pronounced metabolomic changes (Nguyen et al., 2020; Van Thao et al., 2025). In this study, metabolic profiles differed between control and MgCl_2_ pre-treated mussels after live transport, but these differences diminished over the recovery period, indicating partial effectiveness of the pre-treatment. Overall, MgCl_2_ pre-treatment reduced the magnitude of transport-induced metabolic changes in mussels from both families, although it did not fully prevent stress responses. Consistent with this, live transport simulation activated metabolic pathways related to energy metabolism (e.g., GG and APM), as well as immune response and homeostasis (e.g., osmoregulation), reflected by elevated ornithine (May & Rawson, 2023) and itaconate (Delorme et al., 2021; Nguyen et al., 2019). While MgCl_2_ did not fully prevent stress responses, it clearly reduced the magnitude of transport-induced metabolic changes compared with untreated mussels, indicating a partial dampening of stress-related metabolic activity (Cheng et al., 2026).

MgCl_2_ may function as a sedative by lowering mussel activity and metabolic demand. Mg^2+^ ions can disrupt neurotransmission, leading to reduced movement and decreased oxygen consumption, which, in turn, limits reliance on anaerobic metabolism. Elevated Mg^2+^ levels in animal tissues may also increase temperature sensitivity (Frederich et al., 2000). Consequently, low transport temperatures and enhanced Mg^2+^ levels may act synergistically to further suppress mussel metabolism during live transport. Excessive MgCl_2_ concentrations (e.g., 100 g/L), however, may be stressful to mussels, as reflected by a 27-fold increase in succinate levels (Azizan et al., 2021), highlighting the importance of using an appropriate dosage. Similar findings have been reported in other aquaculture species, where pre-treatments decrease but do not eliminate transport stress, and some metabolic adjustments persist post-transport (Kurnaningtyas et al., 2022; Luz & Favero, 2024; Zheng et al., 2023). For example, pharmaceutical pre-treatment in the clam *Ruditapes philippinarum* enhanced resistance to live transport stress, particularly oxidative stress, even if there were still elevated levels of metabolites related to anaerobic metabolism (Zheng et al., 2023).

### Family-specific metabolic responses

Although no mortality occurred, the two selectively-bred mussel families exhibited distinct metabolomic responses during live transport and recovery, indicating different physiological strategies for coping with stress that are likely associated with variation in thermal tolerance and energy allocation mechanisms previously identified in these families (Delorme et al. 2024b). Specifically, the less heat-tolerant (FamC) mussels showing more pathways affected at TP1 and more heat-tolerant (FamF) mussels showing more pathways affected at TP2, likely reflecting inter-familial differences in energetic strategies for stress management (Jiang et al., 2025; Yang et al., 2025).

Less heat-tolerant (FamC) mussels appear to adopt a rapid and metabolically intensive response to transport stress, showing activation of numerous metabolic pathways, such as CC, GDM, GST, CMM and OCP, which not only support energy supply but also fuel other cellular stress responses, such as immune and antioxidation responses (Habte-Tsion et al., 2016; Wu et al., 2021). Such a response mirrors findings from a separate study, where a less heat-tolerant mussel family under heat stress showed greater numbers of differentially regulated genes for cellular stress and immune responses (Ericson et al., 2024). Under stressful conditions, such responses are often accompanied by upregulation of serine, cysteine and methionine, the amino acids critical for antioxidative defences (Martínez et al., 2017; Netto et al., 2007). For instance, in abalone, *Haliotis iris*, aerial exposure associated with live transport has been shown to affect metabolic pathways for energy and amino acid metabolism, accompanied by significant increase in a glycolysis intermediate, phosphoenolpyruvate and free amino acids (FAA), such as valine and tyrosine, which are likely to be involved in osmoregulation and antioxidative response (Alfaro et al., 2021). These responses suggest that live transport triggered early activation of stress-responsive metabolic pathways in FamC mussels, increasing energetic costs and reducing recovery efficiency, consistent with lower stress tolerance. Some stress responses persisting even after one day of recovery despite the MgCl_2_ pre-treatment intended to mitigate transport stress.

In contrast, more heat-tolerant (FamF) mussels, in contrast, may expend less energy during live transport due to an overall decreased metabolism, reflected by fewer differentially altered metabolite levels, in turn, fewer affected pathways, which could be due to a lower number of significantly regulated genes that govern these metabolic activities (Ericson et al., 2024). This energy-saving strategy could delay the activation of multiple stress-response pathways. During recovery the mussels, however, could change strategy, by activating more metabolic pathways to support antioxidation and osmoregulation (Georgoulis et al., 2022). The increased number of enriched pathways after TP2 (1-day recovery), particularly those related to energy metabolism, osmoregulation and antioxidant defence, likely reflects the initiation of energy-demanding protective mechanisms during re-oxygenation after aerial exposure (Fields et al., 2014). This is accompanied by rises in FAA, including alanine, proline, serine and histidine, along with phosphoenolpyruvate, reflecting increased energy demand for synthesising and mobilising these molecular chaperones for supporting protective processes (Goolish & Burton, 1989; Zurburg & De Zwaan, 1981). Similar patterns have been observed in the mussel *Mytilus edulis*, after one day of hypoxia, with elevated levels of FAA (Haider et al., 2020). Consistently, FamF mussels exhibited stronger cellular protective responses, including upregulation of the 70 kDa heat shock protein gene, during recovery after an acute heat challenge (Family F in Delorme et al. 2024b). This delayed but coordinated activation of protective pathways may reflect improved resilience and recovery capacity in more heat-tolerant mussels. Together, these findings suggest that selectively-bred mussel families may employ different metabolic strategies to cope with transport-associated stress. Effectiveness of MgCl_2_ pre-treatment may, therefore, depend partly on the inherent physiological resilience of individual mussel families.

### Implications

The results presented here are consistent with the hypothesis that thermotolerant mussel families may also demonstrate greater tolerance to live transport stress, likely due to their capacity to withstand environmental fluctuations, such as temperature shifts, desiccation and oxygen limitation, underscored by the coordination of different genes and, therefore, metabolic pathways (Ericson et al., 2024). This could also make them desirable candidates for commercial live transport with emersion conditions (Powell et al., 2017). This represents a promising direction for developing aquaculture bivalves with greater tolerance to low-oxygen conditions during live transport. Alternatively, selective breeding programmes can generate genetically distinct families for cultured marine bivalves, such as mussels, oysters and scallops, by selecting individuals from families with superior traits, such as faster growth and better body condition, chosen to establish breeding populations (Nascimento-Schulze et al., 2021; Nguyen et al., 2014). In New Zealand, selectively breeding the green-lipped mussel, *Perna canaliculus*, has been going on for over two decades (Symonds et al., 2018). For instance, 72 selectively-bred families of *P. canaliculus* were tested for tolerance to air exposure to identify the molecular mechanisms underlying this trait (Powell et al., 2017). Mussels with higher tolerance to aerial exposure could, therefore, be ideal candidates for live transport.

Furthermore, when thermally tolerant families are treated with MgCl_2_, their resilience to air exposure can be further enhanced, offering an integrated, possibly integrative strategy to minimise metabolic disruption along the live supply chain, as demonstrated in this study. Tolerance to aerial exposure should, however, not be the sole selection criterion as commercially desirable traits, such as meat: shell ratio and meat quality must also be maintained. Moreover, breeding for thermal tolerance can help the aquaculture industry adapt to increasingly frequent climate-related stress events (Jiang et al., 2025; Sae-Lim et al., 2017). Based on the preliminary, two-family findings of the present study, it therefore appears appropriate to proceed with a broader assessment of *P. canaliculus* from different selectively-bred families to quantify the influence of inter-familial variation on live transport performance and the interaction with MgCl_2_ pre-treatment.

## Conclusions

This study demonstrated the effectiveness of MgCl_2_ pre-treatment on mussels from two different selectively-bred families with contrasting thermal tolerance. This pre-treatment is effective in reducing the stress associated with live transport with family-specific metabolic responses after live transport and recovery. More heat-tolerant mussels also demonstrated higher resistance to transport-induced stress accompanied by more coordinated metabolic adjustments throughout the recovery phase. Based on this initial study using two families, the integration of selective breeding and pre-treatment may interact beneficially to influence the quality of mussels during long-distance live transport. Such integrative strategies can enhance sustainability, animal welfare and product consistency in aquaculture, while mitigating transport-related stress, improving post-transport recovery and ultimately boosting profitability. These strategies may also be transferable to other high-value bivalves, with access to selective breeding programmes, such as Pacific oysters and scallops, which also undergo prolonged live transport. At the same time, the effects of the pre-treatment on mussels’ organoleptic properties, such as texture and taste, require further investigation. Clear guidance on implementing such pre-treatment should also be developed to ensure optimal efficacy. This study establishes a development pipeline for pharmaceutically reducing mussel metabolism during live transport, which can also be adapted for other aquaculture bivalve species.

## Supplementary Information

Below is the link to the electronic supplementary material.

Supplementary Material 1

## Data Availability

The data in this study will be available on reasonable request after permission has been granted from the corresponding author.
