# Supplementary material for: Metabolomic insights into inter-familial and pre-transport treatment effects on live transport stress in selectively-bred mussels, Perna canaliculus
Source: Metabolomics. 2026 Jun 16;22(4):94. doi: 10.1007/s11306-026-02477-7 (PMC13272606; doi:10.1007/s11306-026-02477-7)
Supplement: Supplementary file 1 — Supplementary Material 1 [file 11306_2026_2477_MOESM1_ESM.docx]

**Metabolomic insights into inter-familial and pre-transport treatment effects on live transport stress in selectively-bred mussels, *Perna canaliculus***

Cheng, M.C.F.^1,2*^, Zamora, L.N.^1,2^, Delorme, N.J.^2^, Ragg, N.L.C.^2^, Hickey, A.J.R. ^1^, Dunphy, B.J. ^1^

^1^ School of Biological Sciences, University of Auckland, Private Bag 92019, Auckland, 1142, New Zealand

^2^ Cawthron Institute, Private Bag 2, Nelson 7042, New Zealand

*Email of corresponding author: [cche448@aucklanduni.ac.nz](mailto:cche448@aucklanduni.ac.nz) (M.C.F. Cheng)

**Supporting Information**

This supporting information contains relevant figures and tables cited in the main text.

**Fig. S1** Supervised PLS-DA maximising the distance between control (i.e., mussels directly sampled from the holding tanks) and MgCl_2_ (i.e., mussels experienced MgCl_2_ pre-treatment) groups at different time points (TP1: after aerial exposure at 9 °C for 72 hours to simulate live transport; TP2: after 1-day recovery in seawater; TP3: after 5-day recovery in seawater) for (a) the less heat-tolerant mussel family (FamC), and (b) the more heat-tolerant mussel family (FamF).

**
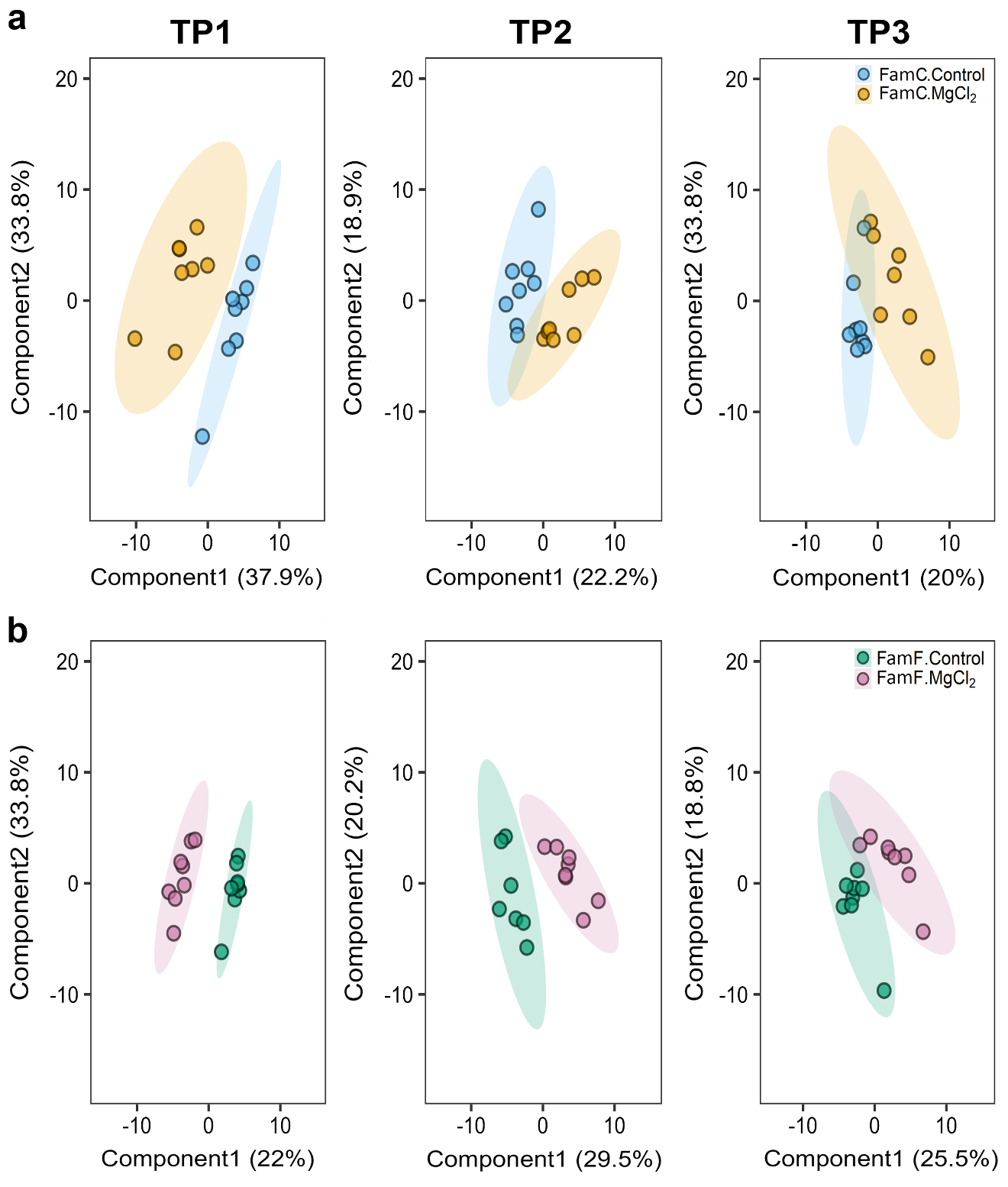
**

**Table S1** The metabolites detected in the mussel gill tissues in the present study, which were used as reference library for pathway analysis (AAD = Amino acid derivative, AAMI = Amino acid metabolic intermediate, NAA = Non-standard amino acid). Metabolites lacking KEGG IDs are often specialised or less common compounds that have not yet been curated in the KEGG database.

| **Category** | **Metabolite** | **KEGG ID** | **Category** | **Metabolite** | **KEGG ID** | **Category** | **Metabolite** | **KEGG ID** |
| --- | --- | --- | --- | --- | --- | --- | --- | --- |
| Amino acid | Alanine (ALA) | C00041 | NAA | gamma-Aminobutyrate (GABA) | C00334 | Fatty acid | Linoleate (LA) | C01595 |
| Amino acid | Asparagine (ASN) | C00152 | NAA | Hydroxyproline (HYP) | C01015 | Fatty acid | Eicosapentaenoate (EPA) | C06428 |
| Amino acid | Aspartate (ASP) | C00049 | NAA | L-threo-3-Hydroxyaspartate (HASP) | C11511 | Fatty acid | Margarate (MA) |  |
| Amino acid | beta-Alanine (BALA) | C00099 | AAD | 4-Hydroxyphenylacetate (PHA) | C00642 | Fatty acid | Myristate (MYRA) | C06424 |
| Amino acid | Cysteine (CYS) | C00097 | AAD | Glutathione (GSH) | C00051 | Fatty acid | Oleate (OA) | C03425 |
| Amino acid | Glutamate (GTA) | C00025 | AAD | Pyroglutamate (PGA) | C01879 | Fatty acid | Palmitelaidate (PE) |  |
| Amino acid | Glutamine (GLN) | C00064 | AAMI | 2-Aminoadipate (AAA) | C00956 | Fatty acid | Palmitate (PA) | C00249 |
| Amino acid | Glycine (GLY) | C00037 | AAMI | Creatinine (CRN) | C00791 | Fatty acid | Palmitoleate (POA) | C08362 |
| Amino acid | Histidine (HIS) | C00135 | AAMI | Cystathionine (CYST) | C02291 | Fatty acid | Pentadecanoate (PDA) | C16537 |
| Amino acid | Homocysteine (HCY) | C00155 | Imino acid | Strombine (STR) | C03790 | Fatty acid | Stearate (SA) | C01530 |
| Amino acid | Isoleucine (ILE) | C00407 | Fatty acid | 10,12-Octadecadienoate (ODA) | C04056 | Fatty acid | Vaccenate (VA) | C08367 |
| Amino acid | Leucine (LEU) | C00123 | Fatty acid | 10,13-dimethyltetradecanoate (DMTDA) |  | Organic acid | *cis*-Aconitate (CAC) | C00417 |
| Amino acid | Lysine (LYS) | C00047 | Fatty acid | 11,14,17-Eicosatrienoate (ETRA) | C16522 | Organic acid | Citrate (CIT) | C00158 |
| Amino acid | Methionine (MET) | C00073 | Fatty acid | 11,14-Eicosadienoate (EDA) | C16525 | Organic acid | Dimethyl aminomalonate (DMAMA) |  |
| Amino acid | Ornithine (ORN) | C00077 | Fatty acid | 11Z-Eicosenoate (ESA) | C16526 | Organic acid | Fumarate (FUM) | C00122 |
| Amino acid | Phenylalanine (PHE) | C00079 | Fatty acid | 13,16-Docosadienoate (DDA) | C16533 | Organic acid | Glutarate (GLTA) | C00489 |
| Amino acid | Proline (PRO) | C00148 | Fatty acid | 9E-Heptadecenoate (HDA) | C16536 | Organic acid | Glyoxylate (GOA) | C00048 |
| Amino acid | Serine (SER) | C00065 | Fatty acid | Adrenate (ADA) | C16527 | Organic acid | Itaconate (ITA) | C00490 |
| Amino acid | Threonine (THR) | C00188 | Fatty acid | alpha-Linolenate (ALLA) | C06427 | Organic acid | Lactate (LAC) | C00256 |
| Amino acid | Tryptophan (TRP) | C00078 | Fatty acid | Arachidonate (ARA) | C00219 | Organic acid | Malate (MAL) | C00149 |
| Amino acid | Tyrosine (TYR) | C00082 | Fatty acid | *cis*-Vaccenate (CVA) | C21944 | Organic acid | Nicotinate (NCA) | C00253 |
| Amino acid | Valine (VAL) | C00183 | Fatty acid | Docosahexaenoate (DHA) | C06429 | Organic acid | Phosphoenolpyruvate (PEP) | C00074 |
| NAA | 1-Aminocyclopropane-1-carboxylate (ACC) | C01234 | Fatty acid | Docosapentaenoate (DPA) | C16513 | Organic acid | Succinate (SUC) | C00042 |
| NAA | 2-Aminobutyrate (AABA) | C02261 | Fatty acid | Dodecanoate (DCA) | C02679 |  |  |  |
| NAA | 3-Aminoisobutyrate (AIBA) | C05145 | Fatty acid | gamma-Linolenate (GLA) | C06426 |  |  |  |

**Table S2** Significantly different metabolites between control and MgCl_2_ pre-treated mussels from (a) the less heat-tolerant family (FamC) and (b) the more heat-tolerant family (FamF) across different sampling time points (i.e., after live transport simulation for 72 hours, and following 1-day and 5-day recovery periods).

| **Metabolite** | ***χ*^2^** | ***df*** | ***p* value** | **Metabolite** | ***χ*^2^** | ***df*** | ***p* value** |
| --- | --- | --- | --- | --- | --- | --- | --- |
| **(a) FamC** |  |  |  | **(b) FamF** |  |  |  |
| 9E-Heptadecenoate | 34.941 | 2 | 2.586‧10^-8^ | 9E-Heptadecenoate | 62.557 | 2 | 2.606‧10^-14^ |
| Methionine | 33.466 | 2 | 5.406‧10^-8^ | Strombine | 59.939 | 2 | 9.650‧10^-14^ |
| Phenylalanine | 24.730 | 2 | 4.266‧10^-6^ | 2-Aminobutyrate | 44.332 | 2 | 2.362‧10^-10^ |
| Ornithine | 21.872 | 2 | 1.780‧10^-5^ | Glutamine | 43.181 | 2 | 4.200‧10^-10^ |
| Strombine | 20.796 | 2 | 3.049‧10^-5^ | 4-Hydroxyphenylacetate | 38.038 | 2 | 5.497‧10^-9^ |
| Asparagine | 19.710 | 2 | 5.248‧10^-5^ | Asparagine | 27.210 | 2 | 1.235‧10^-6^ |
| 4-Hydroxyphenylacetate | 19.506 | 2 | 5.811‧10^-5^ | Proline | 26.879 | 2 | 1.456‧10^-6^ |
| Valine | 17.598 | 2 | 1.509‧10^-4^ | Ornithine | 24.729 | 2 | 4.268‧10^-6^ |
| Glutamine | 17.230 | 2 | 1.814‧10^-4^ | Itaconate | 23.488 | 2 | 7.935‧10^-6^ |
| Tryptophan | 17.158 | 2 | 1.880‧10^-4^ | Cystathionine | 22.492 | 2 | 1.306‧10^-5^ |
| Lysine | 16.428 | 2 | 2.709‧10^-4^ | Fumarate | 20.531 | 2 | 3.482‧10^-5^ |
| Phosphoenolpyruvate | 16.396 | 2 | 2.752‧10^-4^ | Pyroglutamate | 18.964 | 2 | 7.621‧10^-5^ |
| Isoleucine | 15.170 | 2 | 5.081‧10^-4^ | Serine | 15.843 | 2 | 3.628‧10^-4^ |
| Creatinine | 14.680 | 2 | 6.489‧10^-4^ | Threonine | 15.561 | 2 | 4.178‧10^-4^ |
| Cysteine | 14.163 | 2 | 8.405‧10^-4^ | Alanine | 15.046 | 2 | 5.405‧10^-4^ |
| Cystathionine | 13.816 | 2 | 1.000‧10^-3^ | Histidine | 14.873 | 2 | 5.895‧10^-4^ |
| Itaconate | 13.748 | 2 | 1.034‧10^-3^ | Malate | 14.804 | 2 | 6.100‧10^-4^ |
| Leucine | 12.660 | 2 | 1.782‧10^-3^ | gamma-Aminobutyrate | 13.101 | 2 | 1.429‧10^-3^ |
| 2-Aminobutyrate | 10.659 | 2 | 4.845‧10^-3^ | Valine | 13.026 | 2 | 1.484‧10^-3^ |
| Tyrosine | 10.425 | 2 | 5.447‧10^-3^ | 2-Aminoadipate | 11.168 | 2 | 3.758‧10^-3^ |
| 3-Aminoisobutyrate | 10.380 | 2 | 5.571‧10^-3^ | Aconitate | 8.776 | 2 | 1.243‧10^-2^ |
| Malate | 10.228 | 2 | 6.012‧10^-3^ | Isoleucine | 7.683 | 2 | 2.146‧10^-2^ |
| 2-Aminoadipate | 9.544 | 2 | 8.465‧10^-3^ | Creatinine | 7.299 | 2 | 2.600‧10^-2^ |
| Serine | 8.990 | 2 | 1.116‧10^-2^ | Citrate | 7.100 | 2 | 2.873‧10^-2^ |
| Threonine | 8.581 | 2 | 1.370‧10^-2^ | Phosphoenolpyruvate | 6.636 | 2 | 3.623‧10^-2^ |
| Histidine | 7.982 | 2 | 1.848‧10^-2^ |  |  |  |  |
| Glutamate | 7.765 | 2 | 2.060‧10^-2^ |  |  |  |  |
| Citrate | 6.266 | 2 | 4.358‧10^-2^ |  |  |  |  |

**Table S3** Differentially expressed metabolites from the less heat-tolerant family (FamC) at different time points: (a) TP1, after 72-hour aerial exposure at 9 °C for live transport simulation; (b) TP2, after 1-day recovery in seawater, and (c) TP3 after 5-day recovery in seawater for pathway analysis. Metabolites were ordered according to the Benjamini-Hochberg adjusted *p* values (*p_adj_*). |log_2_FC| represents the absolute value of fold change (FC) on a log_2_ scale, and VIP represents variable importance in projection obtained from PLS-DA plot.

| **Metabolite** | ***χ*^2^** | ***df*** | ***p_adj_* value** | **\|log_2_FC\|** | **VIP** | **Metabolite** | ***χ*^2^** | ***df*** | ***p_adj_* value** | **\|log_2_FC\|** | **VIP** |
| --- | --- | --- | --- | --- | --- | --- | --- | --- | --- | --- | --- |
| **(a) TP1** |  |  |  |  |  | **(b) TP2** |  |  |  |  |  |
| Itaconate | 52.918 | 1 | 2.540‧10^-11^ | 2.005 | 1.935 | Serine | 71.048 | 1 | 2.540‧10^-15^ | 0.960 | 2.468 |
| 9E-Heptadecenoate | 27.642 | 1 | 3.550‧10^-6^ | 1.089 | 1.758 | 2-Aminobutyrate | 35.877 | 1 | 7.670‧10^-8^ | 1.262 | 2.180 |
| Methionine | 28.093 | 1 | 3.550‧10^-6^ | 1.177 | 1.752 | Histidine | 19.541 | 1 | 1.198‧10^-4^ | 1.363 | 2.047 |
| Fumarate | 26.688 | 1 | 4.360‧10^-6^ | 1.098 | 1.558 | Threonine | 19.883 | 1 | 1.198‧10^-4^ | 0.894 | 1.905 |
| Histidine | 25.609 | 1 | 6.100‧10^-6^ | 1.246 | 1.733 | Valine | 19.751 | 1 | 1.198‧10^-4^ | 0.877 | 1.918 |
| Strombine | 23.915 | 1 | 1.220‧10^-5^ | 1.502 | 1.709 | Tryptophan | 15.636 | 1 | 8.007‧10^-4^ | 0.672 | 1.499 |
| Valine | 22.721 | 1 | 1.950‧10^-5^ | 0.935 | 1.692 | Itaconate | 15.019 | 1 | 9.713‧10^-4^ | 1.920 | 1.937 |
| Serine | 22.021 | 1 | 2.460‧10^-5^ | 0.686 | 1.677 | Cystathionine | 12.705 | 1 | 2.957‧10^-3^ | 0.905 | 1.596 |
| Tryptophan | 21.758 | 1 | 2.510‧10^-5^ | 1.044 | 1.676 | Hydroxyproline | 10.718 | 1 | 7.043‧10^-3^ | 1.011 | 1.763 |
| Cysteine | 19.941 | 1 | 5.830‧10^-5^ | 0.835 | 1.645 | Proline | 10.868 | 1 | 7.043‧10^-3^ | 0.692 | 1.771 |
| Phenylalanine | 16.625 | 1 | 3.023‧10^-4^ | 0.651 | 1.576 | beta-Alanine | 9.800 | 1 | 1.062‧10^-2^ | 0.699 | 1.716 |
| Phosphoenolpyruvate | 16.378 | 1 | 3.156‧10^-4^ | 1.179 | 1.551 |  |  |  |  |  |  |
| Citrate | 15.682 | 1 | 3.828‧10^-4^ | 0.734 | 1.554 | **(c) TP3** |  |  |  |  |  |
| Isoleucine | 15.468 | 1 | 3.828‧10^-4^ | 0.955 | 1.548 | Glutamine | 20.275 | 1 | 4.896‧10^-4^ | 0.629 | 2.557 |
| Ornithine | 15.527 | 1 | 3.828‧10^-4^ | 1.355 | 1.550 | 2-Aminoadipate | 9.251 | 1 | 3.436‧10^-2^ | 1.069 | 2.001 |
| Threonine | 15.778 | 1 | 3.828‧10^-4^ | 0.717 | 1.556 |  |  |  |  |  |  |
| Malate | 14.129 | 1 | 7.331‧10^-4^ | 0.800 | 1.430 |  |  |  |  |  |  |
| 4-Hydroxyphenylacetate | 11.328 | 1 | 2.933‧10^-3^ | 1.262 | 1.315 |  |  |  |  |  |  |
| Lysine | 9.919 | 1 | 5.970‧10_-3_ | 0.596 | 1.270 |  |  |  |  |  |  |
| Creatinine | 9.393 | 1 | 7.570‧10^-3^ | 1.047 | 1.343 |  |  |  |  |  |  |
| Leucine | 9.186 | 1 | 8.091‧10^-3^ | 0.586 | 1.334 |  |  |  |  |  |  |
| Asparagine | 8.184 | 1 | 1.285‧10^-2^ | 0.615 | 1.285 |  |  |  |  |  |  |
| beta-Alanine | 7.938 | 1 | 1.413‧10^-2^ | 0.705 | 1.220 |  |  |  |  |  |  |
| *cis*-Aconitate | 7.302 | 1 | 1.934‧10^-2^ | 0.634 | 1.236 |  |  |  |  |  |  |
| Glutarate | 6.216 | 1 | 3.424‧10^-2^ | 0.694 | 1.094 |  |  |  |  |  |  |

**Table S4** Differentially expressed metabolites (DEM) from the more heat-tolerant family (FamF) at different time points: (a) TP1, after 72-hour aerial exposure at 9 °C for live transport simulation; and (b) TP2, after 1-day recovery in seawater for pathway analysis. There are no DEM selected in TP3 (after 5-day recovery in seawater) for pathway analysis. Metabolites were ordered according to the Benjamini-Hochberg adjusted *p* values (*p_adj_*). |log_2_FC| represents the absolute value of fold change (FC) on a log_2_ scale, and VIP represents variable importance in projection obtained from PLS-DA plot.

| **Metabolite** | ***χ*^2^** | ***df*** | ***p_adj_* value** | **\|log_2_FC\|** | **VIP** | **Metabolite** | ***χ*^2^** | ***df*** | ***p_adj_* value** | **\|log_2_FC\|** | **VIP** |
| --- | --- | --- | --- | --- | --- | --- | --- | --- | --- | --- | --- |
| **(a) TP1** |  |  |  |  |  | **(b) TP2** |  |  |  |  |  |
| 9E-Heptadecenoate | 67.698 | 1 | 1.391‧10^-14^ | 1.407 | 2.005 | Glutamine | 141.212 | 1 | 1.056‧10^-30^ | 0.957 | 1.977 |
| Phosphoenolpyruvate | 58.217 | 1 | 8.568‧10^-13^ | 0.813 | 1.756 | Valine | 48.016 | 1 | 1.543‧10^-10^ | 1.004 | 1.815 |
| Itaconate | 56.311 | 1 | 1.505‧10^-12^ | 1.919 | 1.967 | 2-Aminobutyrate | 44.787 | 1 | 5.345‧10^-10^ | 1.295 | 1.800 |
| Strombine | 45.831 | 1 | 2.352‧10^-10^ | 2.273 | 1.834 | Histidine | 42.370 | 1 | 1.378‧10^-9^ | 1.425 | 1.787 |
| Glutamine | 37.571 | 1 | 1.287‧10^-8^ | 0.932 | 1.730 | Threonine | 39.356 | 1 | 5.157‧10^-9^ | 0.974 | 1.769 |
| Asparagine | 36.100 | 1 | 2.281‧10^-8^ | 0.946 | 1.802 | Isoleucine | 34.900 | 1 | 4.222‧10^-8^ | 0.755 | 1.739 |
| Histidine | 31.753 | 1 | 1.826‧10^-7^ | 0.982 | 1.818 | Itaconate | 27.589 | 1 | 1.565‧10^-6^ | 2.328 | 1.674 |
| Ornithine | 31.360 | 1 | 1.956‧10^-7^ | 1.229 | 1.777 | Serine | 27.308 | 1 | 1.583‧10^-6^ | 1.304 | 1.671 |
| Tryptophan | 30.076 | 1 | 3.370‧10^-7^ | 0.677 | 1.481 | Asparagine | 24.870 | 1 | 4.974‧10^-6^ | 0.685 | 1.642 |
| Fumarate | 28.041 | 1 | 7.883‧10^-7^ | 1.003 | 1.779 | *cis*-Aconitate | 24.371 | 1 | 5.451‧10^-6^ | 0.969 | 1.636 |
| 4-Hydroxyphenylacetate | 24.136 | 1 | 5.041‧10^-6^ | 2.491 | 1.665 | Proline | 24.307 | 1 | 5.451‧10^-6^ | 0.922 | 1.584 |
| Malate | 15.617 | 1 | 3.539‧10^-4^ | 0.719 | 1.567 | Tryptophan | 23.264 | 1 | 8.589‧10^-6^ | 0.595 | 1.621 |
|  |  |  |  |  |  | Phosphoenolpyruvate | 22.352 | 1 | 1.274‧10^-5^ | 0.984 | 1.142 |
|  |  |  |  |  |  | Alanine | 19.504 | 1 | 4.876‧10^-5^ | 0.788 | 1.563 |
|  |  |  |  |  |  | Citrate | 19.491 | 1 | 4.876‧10^-5^ | 0.806 | 1.563 |
|  |  |  |  |  |  | Cystathionine | 16.017 | 1 | 2.675‧10^-4^ | 1.019 | 1.435 |
|  |  |  |  |  |  | Ornithine | 15.924 | 1 | 2.675‧10^-4^ | 0.696 | 1.492 |
|  |  |  |  |  |  | gamma-Aminobutyrate | 12.709 | 1 | 1.398‧10^-3^ | 0.745 | 1.401 |
|  |  |  |  |  |  | Creatinine | 11.763 | 1 | 2.100‧10^-3^ | 0.768 | 1.379 |
|  |  |  |  |  |  | Succinate | 7.090 | 1 | 2.358‧10^-2^ | 0.675 | 1.178 |
|  |  |  |  |  |  | 2-Aminoadipate | 5.581 | 1 | 4.735‧10^-2^ | 1.068 | 1.083 |

**Table S5** Metabolic pathways identified via pathway analysis of metabolomic data from the less heat-tolerant family (FamC) at different points: (a) TP1, after 72-hour aerial exposure at 9 °C for live transport simulation, (b) TP2, after 1-day recovery in seawater, and (c) TP3, after 5-day recovery in seawater. *Total Cmpd* refers to the total number of compounds detected in this study involved in each pathway; *Hits* indicate the differentially expressed metabolites detected in the identified metabolic pathway; *FDR* represents the false discovery rate.

| **Metabolic pathway** | **Total Cmpd** | **Hits** | ***P* value** | **-log_10_(*p*)** | **FDR** | **Impact** |
| --- | --- | --- | --- | --- | --- | --- |
| **(a) TP1** |  |  |  |  |  |  |
| Citrate cycle (TCA cycle) | 6 | 5 | 1.522‧10^-6^ | 5.818 | 2.892‧10^-5^ | 0.214 |
| Cysteine and methionine metabolism | 6 | 3 | 3.442‧10^-5^ | 4.463 | 2.180‧10^-4^ | 0.387 |
| One carbon pool by folate | 6 | 3 | 3.442‧10^-5^ | 4.463 | 2.180‧10^-4^ | 0.222 |
| Pyruvate metabolism | 4 | 3 | 5.612‧10^-5^ | 4.251 | 2.666‧10^-4^ | 0.040 |
| Glyoxylate and dicarboxylate metabolism | 7 | 4 | 9.496‧10^-5^ | 4.023 | 3.608‧10^-4^ | 0.537 |
| Pantothenate and CoA biosynthesis | 2 | 2 | 2.442‧10^-4^ | 3.612 | 7.734‧10^-4^ | 0.000 |
| Glycine, serine and threonine metabolism | 5 | 3 | 3.860‧10^-4^ | 3.413 | 1.048‧10^-3^ | 0.326 |
| Sphingolipid metabolism | 1 | 1 | 6.447‧10^-4^ | 3.191 | 1.370‧10^-3^ | 0.000 |
| Tryptophan metabolism | 1 | 1 | 6.491‧10^-4^ | 3.188 | 1.370‧10^-3^ | 0.235 |
| Glutathione metabolism | 5 | 1 | 9.310‧10^-4^ | 3.031 | 1.474‧10^-3^ | 0.011 |
| Taurine and hypotaurine metabolism | 1 | 1 | 9.310‧10^-4^ | 3.031 | 1.474‧10^-3^ | 0.000 |
| Thiamine metabolism | 1 | 1 | 9.310‧10^-4^ | 3.031 | 1.474‧10^-3^ | 0.000 |
| Phenylalanine metabolism | 1 | 1 | 1.898‧10^-3^ | 2.722 | 2.445‧10^-3^ | 1.000 |
| Phenylalanine, tyrosine and tryptophan biosynthesis | 2 | 1 | 1.898‧10^-3^ | 2.722 | 2.445‧10^-3^ | 0.000 |
| Alanine, aspartate and glutamate metabolism | 6 | 1 | 2.272‧10^-3^ | 2.644 | 2.445‧10^-3^ | 0.000 |
| Tyrosine metabolism | 2 | 1 | 2.272‧10^-3^ | 2.644 | 2.445‧10^-3^ | 0.000 |
| Glycolysis or Gluconeogenesis | 1 | 1 | 2.428‧10^-3^ | 2.615 | 2.445‧10^-3^ | 0.101 |
| Arginine and proline metabolism | 4 | 1 | 2.445‧10^-3^ | 2.612 | 2.445‧10^-3^ | 0.148 |
| Arginine biosynthesis | 3 | 1 | 2.445‧10^-3^ | 2.612 | 2.445‧10^-3^ | 0.143 |
| **(b) TP2** | |  |  |  |  |  |
| Sphingolipid metabolism | 1 | 1 | 4.805‧10^-6^ | 5.318 | 1.922‧10^-5^ | 0.000 |
| Glyoxylate and dicarboxylate metabolism | 7 | 1 | 4.805‧10^-6^ | 5.318 | 1.922‧10^-5^ | 0.000 |
| Glycine, serine and threonine metabolism | 5 | 3 | 1.628‧10^-3^ | 2.788 | 3.851‧10^-3^ | 0.326 |
| One carbon pool by folate | 6 | 2 | 2.407‧10^-3^ | 2.619 | 3.851‧10^-3^ | 0.398 |
| Cysteine and methionine metabolism | 6 | 2 | 2.407‧10^-3^ | 2.619 | 3.851‧10^-3^ | 0.366 |
| Arginine and proline metabolism | 4 | 1 | 8.090‧10^-3^ | 2.092 | 1.079‧10^-2^ | 0.082 |
| Pantothenate and CoA biosynthesis | 2 | 1 | 1.101‧10^-2^ | 1.958 | 1.258‧10^-2^ | 0.000 |
| Tryptophan metabolism | 1 | 1 | 3.140‧10^-2^ | 1.503 | 3.140‧10^-2^ | 0.235 |
| **(c) TP3** | | | | | | |
| Arginine biosynthesis | 3 | 1 | 1.056‧10^-3^ | 2.977 | 1.056‧10^-3^ | 0.214 |
| Alanine, aspartate and glutamate metabolism | 6 | 1 | 1.056‧10^-3^ | 2.977 | 1.056‧10^-3^ | 0.196 |
| Pyrimidine metabolism | 1 | 1 | 1.056‧10^-3^ | 2.977 | 1.056‧10^-3^ | 0.000 |
| Glyoxylate and dicarboxylate metabolism | 7 | 1 | 1.056‧10^-3^ | 2.977 | 1.056‧10^-3^ | 0.000 |
| Nitrogen metabolism | 2 | 1 | 1.056‧10^-3^ | 2.977 | 1.056‧10^-3^ | 0.000 |

**Table S6** Metabolic pathways identified via pathway analysis of metabolomic data from the more heat-tolerant family (FamF) at different points: (a) TP1, after 72-hour aerial exposure at 9 °C for live transport simulation, and (b) TP2, after 1-day recovery in seawater. There are no differentially expressed metabolites selected in TP3 (after 5-day recovery in seawater) for pathway analysis. *Total Cmpd* refers to the total number of compounds detected in this study involved in each pathway; *Hits* indicate the differentially expressed metabolites detected in the identified metabolic pathway; *FDR* represents the false discovery rate.

| **Metabolic pathway** | **Total Cmpd** | **Hits** | ***P* value** | **-log_10_(*p*)** | **FDR** | **Impact** |
| --- | --- | --- | --- | --- | --- | --- |
| **(a) TP1** |  |  |  |  |  |  |
| Arginine biosynthesis | 3 | 2 | 2.755‧10^-6^ | 5.560 | 3.030‧10^-5^ | 0.357 |
| Alanine, aspartate and glutamate metabolism | 6 | 2 | 9.075‧10^-6^ | 5.042 | 3.643‧10^-5^ | 0.196 |
| Glyoxylate and dicarboxylate metabolism | 7 | 2 | 1.589‧10^-5^ | 4.799 | 3.643‧10^-5^ | 0.000 |
| Citrate cycle (TCA cycle) | 6 | 3 | 1.656‧10^-5^ | 4.781 | 3.643‧10^-5^ | 0.074 |
| Pyruvate metabolism | 4 | 3 | 1.656‧10^-5^ | 4.781 | 3.643‧10^-5^ | 0.040 |
| Tyrosine metabolism | 2 | 1 | 2.121‧10^-4^ | 3.673 | 3.441‧10^-4^ | 0.000 |
| Arginine and proline metabolism | 4 | 1 | 2.189‧10^-4^ | 3.660 | 3.441‧10^-4^ | 0.148 |
| Glycolysis or Gluconeogenesis | 1 | 1 | 2.934‧10^-4^ | 3.533 | 4.034‧10^-4^ | 0.101 |
| Pyrimidine metabolism | 1 | 1 | 4.091‧10^-4^ | 3.388 | 4.500‧10^-4^ | 0.000 |
| Nitrogen metabolism | 2 | 1 | 4.091‧10^-4^ | 3.388 | 4.500‧10^-4^ | 0.000 |
| Tryptophan metabolism | 1 | 1 | 5.006‧10^-3^ | 2.301 | 5.006‧10^-3^ | 0.235 |
| **(b) TP2** | |  |  |  |  |  |
| Pyrimidine metabolism | 1 | 1 | 5.508‧10^-8^ | 7.259 | 3.277‧10^-7^ | 0.000 |
| Nitrogen metabolism | 2 | 1 | 5.508‧10^-8^ | 7.259 | 3.277‧10^-7^ | 0.000 |
| Arginine biosynthesis | 3 | 2 | 6.143‧10^-8^ | 7.212 | 3.277‧10^-7^ | 0.357 |
| Alanine, aspartate and glutamate metabolism | 6 | 3 | 8.213‧10^-8^ | 7.086 | 3.285‧10^-7^ | 0.196 |
| Glyoxylate and dicarboxylate metabolism | 7 | 4 | 1.189‧10^-5^ | 4.925 | 3.803‧10^-5^ | 0.537 |
| Glycine, serine and threonine metabolism | 5 | 3 | 2.799‧10^-5^ | 4.553 | 7.465‧10^-5^ | 0.326 |
| One carbon pool by folate | 6 | 2 | 5.000‧10^-5^ | 4.301 | 1.000‧10^-4^ | 0.398 |
| Cysteine and methionine metabolism | 6 | 2 | 5.000‧10^-5^ | 4.301 | 1.000‧10^-4^ | 0.366 |
| Arginine and proline metabolism | 4 | 3 | 1.435‧10^-4^ | 3.843 | 2.551‧10^-4^ | 0.262 |
| Sphingolipid metabolism | 1 | 1 | 3.088‧10^-4^ | 3.510 | 4.941‧10^-4^ | 0.000 |
| Citrate cycle (TCA cycle) | 6 | 4 | 5.082‧10^-4^ | 3.294 | 7.391‧10^-4^ | 0.173 |
| Tryptophan metabolism | 1 | 1 | 6.080‧10^-4^ | 3.216 | 8.107‧10^-4^ | 0.235 |
| Propanoate metabolism | 1 | 1 | 2.767‧10^-2^ | 1.558 | 3.163‧10^-2^ | 0.000 |
| Butanoate metabolism | 1 | 1 | 2.767‧10^-2^ | 1.558 | 3.163‧10^-2^ | 0.000 |
| Glycolysis or Gluconeogenesis | 1 | 1 | 3.400‧10^-2^ | 1.469 | 3.400‧10^-2^ | 0.101 |
| Pyruvate metabolism | 4 | 1 | 3.400‧10^-2^ | 1.469 | 3.400‧10^-2^ | 0.000 |
